# Outbreak of Monkeypox in South East Asia; Spotlight on Bangladesh, Pakistan and India

**DOI:** 10.1016/j.amsu.2022.104361

**Published:** 2022-09-08

**Authors:** Govinda Khatri, Syeda Lamiya Mir, Priya, Mohammad Mehedi Hasan

**Affiliations:** a Dow University of Health Sciences, Karachi, Pakistan; b Department of Biochemistry and Molecular Biology, Faculty of Life Science, Mawlana Bhashani Science and Technology University, Tangail, Bangladesh

Dear Editor,

Monkeypox, an unusual viral disease that affects both humans and animals, has long been prominent in portions of Central and West Africa; it was first found in humans in 1970. The symptoms caused by Monkeypox have been shown in Fig. 1. According to the World Health Organization, the monkeypox virus is prevalent in 12 endemic countries (WHO), however, twelve non-endemic WHO member countries have lately reported newly found monkeypox virus infections [1]. The first incidence of monkeypox in Southeast Asia was reported in Singapore; the patient was a 42-year-old British man who works as a flight attendant and had traveled into and out of Singapore; he tested positive on June 20 [2].

**Figure-1:**
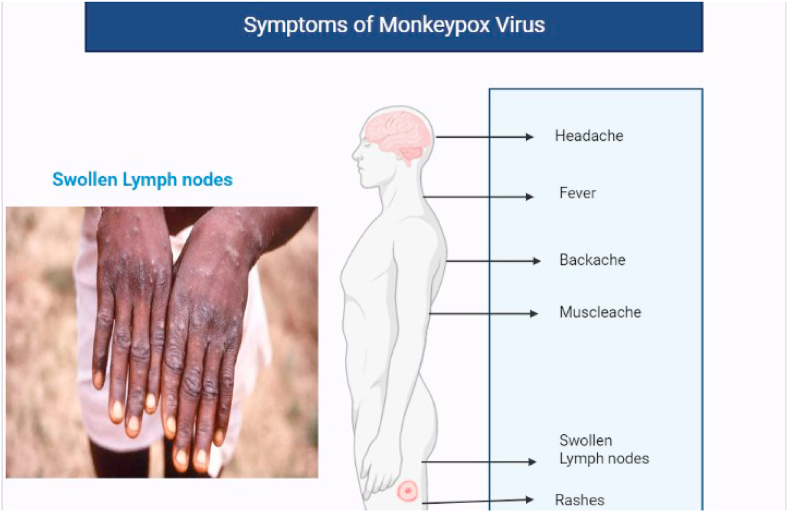
Symptoms of Monkeypox virus.

In response to the global spread of monkeypox, Bangladesh has become the first country to put restrictions on sailors. Despite the fact that no cases have been confirmed in Bangladesh, the government has issued a health warning owing to the spread of the monkeypox virus in other areas of the world. Unless there is an emergency, all crew members are not authorized to utilize shore permits at Chittagong Port, and signed-off crew members are subject to health examinations. Crew changes, port entry, and developing vaccination restrictions remain major worldwide issues for mariners post-COVID-19. As the world prepares for the disease's spread, neighboring nations such as China and India have discussed strengthening travel restrictions [3].

Although there hasn't been a confirmed case of monkeypox in Pakistan, the virus will almost certainly spread there. Given the burden the COVID-19 pandemic has had on Pakistan's already-struggling healthcare sector, several preventative measures must be implemented to halt the virus's eradication [4]. If monkeypox spreads, the failing healthcare system will be brought to its knees. Because Pakistan lacks a virus diagnostic facility, the government of health has determined that samples may be transferred overseas for testing in an emergency.

The first suspected case of monkeypox in India is a 5-year-old boy from Ghaziabad, Uttar Pradesh. Although the monkeypox virus has not yet been found in India, a sample from a suspected monkeypox case in Ghaziabad has been submitted for testing [5].

Due to a lack of knowledge about the current monkeypox virus epidemic, the World Health Organization (WHO) is collaborating with affected countries and others to improve disease surveillance in order to identify and assist people who may be affected, as well as to provide information on how to manage the disease. Until then, nations are advised to follow current WHO guidelines. Furthermore, COVID-19 and monkeypox can coexist despite being from distinct viral families. The pandemic COVID-19 virus can make the body more vulnerable, increasing the likelihood of mortality in a cohabitation setting. Because of the failing economies and bad quality of the healthcare system caused by COVID-19, low- and middle-income nations are more likely to face further suffering.

## Ethics statement

The present study includes printed and published information; therefore, formal ethical clearance was not applicable for this study.

## Sources of funding

N/A.

## Author contributions

All authors meet the inclusion criteria, and all authors read and approved the final version of the manuscript.

## Registration of research studies

1. Name of the registry: N/A.

2. Unique Identifying number or registration ID: N/A.

3. Hyperlink to your specific registration (must be publicly accessible and will be checked): N/A.

## Guarantor

Mohammad Mehedi HasanDepartment of Biochemistry and Molecular Biology, Faculty of Life Science, Mawlana BhashaniScience and Technology University, Tangail, 1902, Bangladesh. Email: mehedi.bmb.mbstu@gmail.com.

## Consent

N/A.

## Declaration of competing interest

The authors declare that there is no conflict of interest.
